# 7-Hy­droxy-4-methyl-8-(3-methyl­benzo­yl)-2*H*-chromen-2-one ethanol monosolvate

**DOI:** 10.1107/S1600536811046630

**Published:** 2011-11-09

**Authors:** Shu-Ping Yang, Li-Jun Han, Xiao-Yun Chen, Zhuan Gao

**Affiliations:** a College of Chemical Engineering, Huaihai Institute of Technology, Lianyungang 222005, People’s Republic of China; b College of Mathematics and Science, Huaihai Institute of Technology, Lianyungang 222005, People’s Republic of China

## Abstract

In the title compound, C_18_H_14_O_4_·C_2_H_6_O, the coumarin ring system is approximately planar with a maximum deviation of 0.037 (3) Å and is nearly perpendicular to the benzene ring, making a dihedral angle of 86.55 (9)°. In the crystal, mol­ecules are linked by classical O—H⋯O hydrogen bonds and weak C—H⋯O inter­actions.

## Related literature

For the biological activity of coumarins, see: Sharma *et al.* (2005 ▶); Xiao *et al.* (2010 ▶); Iqbal *et al.* (2009 ▶); Siddiqui *et al.* (2009 ▶); Rollinger *et al.* (2004 ▶); Brühlmann *et al.* (2001 ▶). For a related structure, see: Yang *et al.* (2010 ▶).
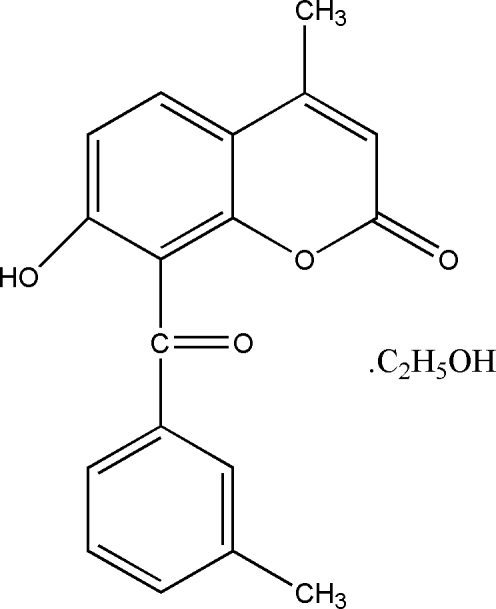


## Experimental

### 

#### Crystal data

C_18_H_14_O_4_·C_2_H_6_O
*M* *_r_* = 340.36
Monoclinic, 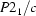

*a* = 12.4562 (6) Å
*b* = 10.0341 (5) Å
*c* = 14.8999 (7) Å
β = 111.980 (3)°
*V* = 1726.93 (14) Å^3^
*Z* = 4
Mo *K*α radiation
μ = 0.09 mm^−1^
*T* = 298 K
0.30 × 0.25 × 0.20 mm

#### Data collection

Bruker APEXII CCD area-detector diffractometer
Absorption correction: multi-scan (*SADABS*; Bruker, 2001 ▶) *T*_min_ = 0.972, *T*_max_ = 0.982
12216 measured reflections
3021 independent reflections
1762 reflections with *I* > 2σ(*I*)
*R*_int_ = 0.119

#### Refinement

*R*[*F* ^2^ > 2σ(*F* ^2^)] = 0.057
*wR*(*F* ^2^) = 0.188
*S* = 1.05
3021 reflections
229 parameters
H-atom parameters constrained
Δρ_max_ = 0.19 e Å^−3^
Δρ_min_ = −0.25 e Å^−3^

### 

Data collection: *APEX2* (Bruker, 2007 ▶); cell refinement: *SAINT* (Bruker, 2007 ▶); data reduction: *SAINT*; program(s) used to solve structure: *SHELXS97* (Sheldrick, 2008 ▶); program(s) used to refine structure: *SHELXL97* (Sheldrick, 2008 ▶); molecular graphics: *DIAMOND* (Brandenburg & Berndt, 1999 ▶); software used to prepare material for publication: *SHELXL97*.

## Supplementary Material

Crystal structure: contains datablock(s) I, global. DOI: 10.1107/S1600536811046630/xu5373sup1.cif

Structure factors: contains datablock(s) I. DOI: 10.1107/S1600536811046630/xu5373Isup2.hkl

Supplementary material file. DOI: 10.1107/S1600536811046630/xu5373Isup3.cml

Additional supplementary materials:  crystallographic information; 3D view; checkCIF report

## Figures and Tables

**Table 1:** Hydrogen-bond geometry (Å, °)

*D*—H⋯*A*	*D*—H	H⋯*A*	*D*⋯*A*	*D*—H⋯*A*
O3—H3⋯O5^i^	0.82	1.82	2.629 (3)	166
O5—H5*A*⋯O2	0.82	1.95	2.764 (3)	169
C17—H17⋯O2^ii^	0.93	2.54	3.398 (4)	154
C20—H20*B*⋯O4^iii^	0.96	2.53	3.489 (5)	177

Symmetry codes: (i) 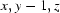; (ii) 
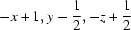
; (iii) 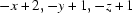.

**Fig. 1.**
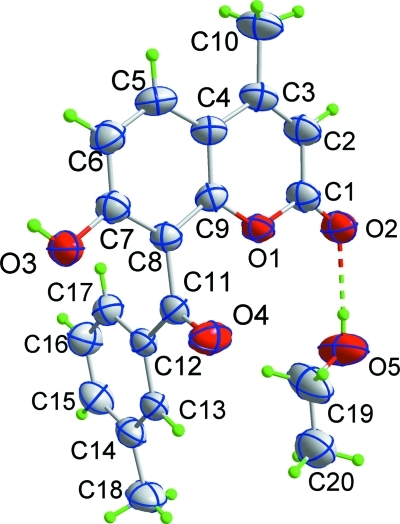
The asymmetric unit of title structure, showing 50% probability displacement ellipsoids for non-H atoms and the atom-numbering scheme, intramolecular O—H···O contact is shown.

**Fig. 2.**
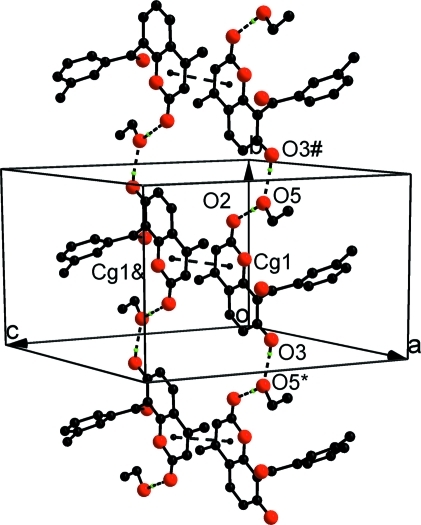
The molecular doubled chain of R44(22) ring parallel to the [010] direction. [Symmetry codes: (*) x, -1 + y, z; (#) x, 1 + y, 1 - z;(&)1 - x, 1 - y, 1 - z].

## supplementary crystallographic information

### Comment

Coumarins are very well known for their biological activity, such as antioxidants (Sharma *et al.*, 2005), anticancer activity (Xiao *et al.*, 2010), antiamoebic (Iqbal *et al.*, 2009), anticonvulsant activity (Siddiqui *et al.*, 2009) and inhibitions of acetylcholinesterase and monoamine oxidase (Rollinger *et al.*, 2004; Brühlmann *et al.*, 2001). Previous we have decribed the crystal structure of 8-benzoyl-7-hydroxy-4-methyl coumarin (Yang *et al.*, 2010). As part of our study of the crystal structures of coumarin derivatives with 7-hydroxy, we report here the crystal structure of 8-(3-methylbenzoyl)-7-hydroxy-4-methyl-2*H*-1-benzopyran-2-one, (I).

In the molecule (I), the asymmetric unit contains one coumarin molecule and one ethanol molecule, and which are linked together by one O—H···O hydrogen bond (Table 1 and Fig. 1). The coumarin moiety (r.m.s deviations 0.0214 Å) and phenyl ring are perpendicular to each other with a dihedral angle of 86.55 (9)° between the plane of the atoms O1—O3/C1—C9 and the plane of C12—C17.

In crystal structure of (I), atom O3 in the molecule at (*x*, *y*, *z*) acts as hydrogen bond donor to atom O5 in the molecule at (*x*, *y* - 1, *z*), forming a *C*(10) chain running parallel to the [010] direction and generated by translation. Inversionally related molecular chains are linked together by a weak π–π interaction, the ring centroid *Cg*1[O1/C1—C4/C9] in the molecule at (*x*, *y*, *z*) connects *Cg*1 in the molecule at (1 - *x*, 1 - *y*, 1 - *z*) [centroid–centroid distance = 3.57278 (17) Å], so forming a doubled chain of *R*_4_^4^(22) ring parallel to the [010] direction (Fig. 2). Neighboring doubled chains are linked into three-dimensional crystal structure by weak C—H···O hydrogen bonds (Table.1).

### Experimental

The mixture containing 1.47 g (5 mmol) of dry powdered 7-(3-methylbenzoxy)-4-methylcoumarin and 2.0 g (15 mmol) of anhydrous aluminium chloride was heated for about 2 h at 463 K in an oil bath, then 30 ml of dilute (1:7) hydrochloric acid is added and the mixture is heated on a steam bath for 60 min, the crude products were filtered off, washed with water. Single crystals of (I) suitable for X-ray structure analysis were obtained by recrystallizing the crude product from a 95% ethanol solution, m.p. 503–504 K.

### Refinement

H atoms were placed in calculated positions with O—H = 0.82 Å (hydroxyl), C—H = 0.93 (aromatic), 0.96 (methyl) and 0.97 Å (methylene), and refined in riding mode with *U*_iso_(H) = 1.2*U*_eq_(C) (aromatic and methylene) and *U*_iso_(H) = 1.5*U*_eq_(C,O) (methyl and hydroxyl).

### Crystal data

**Table:**

C_18_H_14_O_4_·C_2_H_6_O	*F*(000) = 720
*M**_r_* = 340.36	*D*_x_ = 1.309 Mg m^−^^3^
Monoclinic, *P*2_1_/*c*	Mo *K*α radiation, λ = 0.71073 Å
Hall symbol: -P 2ybc	Cell parameters from 1087 reflections
*a* = 12.4562 (6) Å	θ = 2.5–27.8°
*b* = 10.0341 (5) Å	µ = 0.09 mm^−^^1^
*c* = 14.8999 (7) Å	*T* = 298 K
β = 111.980 (3)°	Prism, colourless
*V* = 1726.93 (14) Å^3^	0.30 × 0.25 × 0.20 mm
*Z* = 4	

### Data collection

**Table:**

Bruker APEXII CCD area-detector diffractometer	3021 independent reflections
Radiation source: fine-focus sealed tube	1762 reflections with *I* > 2σ(*I*)
graphite	*R*_int_ = 0.119
φ and ω scans	θ_max_ = 25.0°, θ_min_ = 1.8°
Absorption correction: multi-scan (*SADABS*; Bruker, 2001)	*h* = −14→8
*T*_min_ = 0.972, *T*_max_ = 0.982	*k* = −11→8
12216 measured reflections	*l* = −15→17

### Refinement

**Table:**

Refinement on *F*^2^	Primary atom site location: structure-invariant direct methods
Least-squares matrix: full	Secondary atom site location: difference Fourier map
*R*[*F*^2^ > 2σ(*F*^2^)] = 0.057	Hydrogen site location: inferred from neighbouring sites
*wR*(*F*^2^) = 0.188	H-atom parameters constrained
*S* = 1.05	*w* = 1/[σ^2^(*F*_o_^2^) + (0.0767*P*)^2^ + 0.8164*P*] where *P* = (*F*_o_^2^ + 2*F*_c_^2^)/3
3021 reflections	(Δ/σ)_max_ < 0.001
229 parameters	Δρ_max_ = 0.19 e Å^−^^3^
0 restraints	Δρ_min_ = −0.25 e Å^−^^3^

### Special details

**Table:**

Geometry. All e.s.d.'s (except the e.s.d. in the dihedral angle between two l.s. planes) are estimated using the full covariance matrix. The cell e.s.d.'s are taken into account individually in the estimation of e.s.d.'s in distances, angles and torsion angles; correlations between e.s.d.'s in cell parameters are only used when they are defined by crystal symmetry. An approximate (isotropic) treatment of cell e.s.d.'s is used for estimating e.s.d.'s involving l.s. planes.
Refinement. Refinement of *F*^2^ against ALL reflections. The weighted *R*-factor *wR* and goodness of fit *S* are based on *F*^2^, conventional *R*-factors *R* are based on *F*, with *F* set to zero for negative *F*^2^. The threshold expression of *F*^2^ > σ(*F*^2^) is used only for calculating *R*-factors(gt) *etc*. and is not relevant to the choice of reflections for refinement. *R*-factors based on *F*^2^ are statistically about twice as large as those based on *F*, and *R*- factors based on ALL data will be even larger.

### Fractional atomic coordinates and isotropic or equivalent isotropic displacement parameters (Å^2^)

**Table:**

	*x*	*y*	*z*	*U*_iso_*/*U*_eq_	
C1	0.5042 (3)	0.6046 (3)	0.3838 (2)	0.0487 (8)	
C2	0.3975 (3)	0.5458 (3)	0.3794 (2)	0.0547 (9)	
H2	0.3386	0.6016	0.3809	0.066*	
C3	0.3789 (3)	0.4140 (3)	0.3734 (2)	0.0498 (8)	
C4	0.4702 (2)	0.3271 (3)	0.3715 (2)	0.0439 (7)	
C5	0.4632 (3)	0.1883 (3)	0.3652 (2)	0.0538 (8)	
H5	0.3943	0.1467	0.3595	0.065*	
C6	0.5548 (3)	0.1119 (3)	0.3673 (2)	0.0523 (8)	
H6	0.5474	0.0197	0.3626	0.063*	
C7	0.6596 (3)	0.1722 (3)	0.3765 (2)	0.0460 (8)	
C8	0.6708 (2)	0.3099 (3)	0.38248 (19)	0.0395 (7)	
C9	0.5751 (2)	0.3841 (3)	0.37894 (18)	0.0400 (7)	
C10	0.2669 (3)	0.3553 (4)	0.3712 (3)	0.0717 (11)	
H10A	0.2202	0.4242	0.3828	0.108*	
H10B	0.2828	0.2882	0.4205	0.108*	
H10C	0.2261	0.3159	0.3090	0.108*	
C11	0.7839 (2)	0.3780 (3)	0.3970 (2)	0.0409 (7)	
C12	0.8089 (2)	0.4124 (3)	0.31036 (19)	0.0389 (7)	
C13	0.9089 (2)	0.4854 (3)	0.3207 (2)	0.0445 (7)	
H13	0.9614	0.5062	0.3823	0.053*	
C14	0.9305 (3)	0.5269 (3)	0.2408 (2)	0.0524 (8)	
C15	0.8528 (3)	0.4895 (4)	0.1505 (3)	0.0639 (10)	
H15	0.8668	0.5149	0.0959	0.077*	
C16	0.7554 (3)	0.4160 (4)	0.1391 (2)	0.0637 (10)	
H16	0.7050	0.3917	0.0775	0.076*	
C17	0.7327 (3)	0.3784 (3)	0.2191 (2)	0.0489 (8)	
H17	0.6661	0.3301	0.2115	0.059*	
C18	1.0332 (3)	0.6147 (4)	0.2514 (3)	0.0744 (11)	
H18A	1.0066	0.7024	0.2278	0.112*	
H18B	1.0751	0.5776	0.2147	0.112*	
H18C	1.0833	0.6197	0.3184	0.112*	
O1	0.59052 (16)	0.51973 (19)	0.38378 (14)	0.0455 (5)	
O2	0.52656 (19)	0.7229 (2)	0.38776 (17)	0.0622 (7)	
O3	0.75312 (19)	0.1018 (2)	0.37973 (17)	0.0611 (6)	
H3	0.7378	0.0220	0.3759	0.092*	
O4	0.84811 (18)	0.4066 (2)	0.47834 (15)	0.0611 (7)	
C19	0.8207 (4)	0.7597 (4)	0.4184 (4)	0.0976 (15)	
H19A	0.8036	0.6926	0.3680	0.117*	
H19B	0.8307	0.7144	0.4785	0.117*	
C20	0.9288 (4)	0.8253 (4)	0.4285 (4)	0.0938 (14)	
H20A	0.9239	0.8584	0.3667	0.141*	
H20B	0.9914	0.7626	0.4523	0.141*	
H20C	0.9425	0.8981	0.4732	0.141*	
O5	0.7296 (2)	0.8436 (2)	0.3963 (3)	0.1013 (11)	
H5A	0.6743	0.8040	0.4008	0.152*	

### Atomic displacement parameters (Å^2^)

**Table:**

	*U*^11^	*U*^22^	*U*^33^	*U*^12^	*U*^13^	*U*^23^
C1	0.0388 (18)	0.049 (2)	0.0555 (19)	0.0084 (15)	0.0140 (14)	0.0061 (15)
C2	0.0376 (18)	0.060 (2)	0.065 (2)	0.0087 (16)	0.0180 (15)	0.0060 (17)
C3	0.0358 (17)	0.062 (2)	0.0499 (18)	−0.0011 (16)	0.0140 (13)	0.0072 (15)
C4	0.0359 (16)	0.049 (2)	0.0452 (17)	−0.0037 (14)	0.0129 (13)	0.0039 (14)
C5	0.0417 (18)	0.058 (2)	0.061 (2)	−0.0136 (16)	0.0182 (15)	0.0034 (16)
C6	0.052 (2)	0.0435 (19)	0.062 (2)	−0.0063 (16)	0.0216 (16)	0.0020 (15)
C7	0.0421 (18)	0.0450 (19)	0.0511 (18)	0.0015 (15)	0.0177 (14)	0.0041 (14)
C8	0.0384 (16)	0.0392 (17)	0.0408 (16)	−0.0007 (13)	0.0148 (12)	0.0016 (13)
C9	0.0401 (17)	0.0388 (17)	0.0398 (15)	−0.0019 (14)	0.0135 (13)	0.0038 (13)
C10	0.041 (2)	0.085 (3)	0.092 (3)	−0.0007 (19)	0.0287 (18)	0.011 (2)
C11	0.0358 (16)	0.0378 (17)	0.0459 (17)	0.0044 (13)	0.0117 (14)	0.0010 (13)
C12	0.0319 (15)	0.0361 (16)	0.0486 (16)	0.0030 (13)	0.0149 (13)	−0.0006 (13)
C13	0.0358 (16)	0.0409 (18)	0.0566 (18)	0.0009 (13)	0.0170 (14)	−0.0052 (14)
C14	0.050 (2)	0.0468 (19)	0.069 (2)	0.0021 (15)	0.0332 (17)	0.0032 (16)
C15	0.067 (2)	0.075 (3)	0.060 (2)	0.004 (2)	0.0343 (19)	0.0069 (18)
C16	0.058 (2)	0.083 (3)	0.0475 (19)	−0.005 (2)	0.0169 (16)	−0.0057 (17)
C17	0.0451 (18)	0.0514 (19)	0.0493 (18)	−0.0030 (15)	0.0167 (14)	−0.0027 (14)
C18	0.063 (2)	0.071 (3)	0.103 (3)	−0.010 (2)	0.048 (2)	0.005 (2)
O1	0.0357 (11)	0.0407 (12)	0.0602 (13)	0.0028 (9)	0.0179 (10)	0.0051 (9)
O2	0.0485 (14)	0.0451 (14)	0.0929 (18)	0.0074 (11)	0.0262 (12)	0.0043 (12)
O3	0.0509 (14)	0.0423 (13)	0.0938 (17)	0.0020 (11)	0.0314 (12)	−0.0004 (12)
O4	0.0472 (14)	0.0809 (17)	0.0482 (13)	−0.0111 (12)	0.0098 (11)	−0.0003 (11)
C19	0.097 (3)	0.059 (3)	0.171 (5)	0.012 (3)	0.088 (3)	0.017 (3)
C20	0.071 (3)	0.081 (3)	0.131 (4)	0.006 (2)	0.040 (3)	−0.009 (3)
O5	0.0615 (17)	0.0419 (15)	0.203 (3)	0.0025 (13)	0.052 (2)	0.0129 (17)

### Geometric parameters (Å, °)

**Table:**

C1—O2	1.216 (4)	C12—C17	1.379 (4)
C1—O1	1.372 (4)	C12—C13	1.403 (4)
C1—C2	1.433 (4)	C13—C14	1.379 (4)
C2—C3	1.340 (4)	C13—H13	0.9300
C2—H2	0.9300	C14—C15	1.384 (5)
C3—C4	1.441 (4)	C14—C18	1.512 (4)
C3—C10	1.503 (4)	C15—C16	1.375 (5)
C4—C9	1.393 (4)	C15—H15	0.9300
C4—C5	1.397 (4)	C16—C17	1.376 (4)
C5—C6	1.364 (4)	C16—H16	0.9300
C5—H5	0.9300	C17—H17	0.9300
C6—C7	1.399 (4)	C18—H18A	0.9600
C6—H6	0.9300	C18—H18B	0.9600
C7—O3	1.348 (3)	C18—H18C	0.9600
C7—C8	1.387 (4)	O3—H3	0.8200
C8—C9	1.390 (4)	C19—O5	1.352 (5)
C8—C11	1.507 (4)	C19—C20	1.454 (6)
C9—O1	1.372 (3)	C19—H19A	0.9700
C10—H10A	0.9600	C19—H19B	0.9700
C10—H10B	0.9600	C20—H20A	0.9600
C10—H10C	0.9600	C20—H20B	0.9600
C11—O4	1.211 (3)	C20—H20C	0.9600
C11—C12	1.478 (4)	O5—H5A	0.8200
			
O2—C1—O1	116.1 (3)	C13—C12—C11	119.7 (2)
O2—C1—C2	126.6 (3)	C14—C13—C12	121.0 (3)
O1—C1—C2	117.3 (3)	C14—C13—H13	119.5
C3—C2—C1	122.9 (3)	C12—C13—H13	119.5
C3—C2—H2	118.5	C13—C14—C15	117.8 (3)
C1—C2—H2	118.5	C13—C14—C18	121.1 (3)
C2—C3—C4	118.7 (3)	C15—C14—C18	121.1 (3)
C2—C3—C10	121.6 (3)	C16—C15—C14	122.0 (3)
C4—C3—C10	119.7 (3)	C16—C15—H15	119.0
C9—C4—C5	116.6 (3)	C14—C15—H15	119.0
C9—C4—C3	118.3 (3)	C15—C16—C17	119.8 (3)
C5—C4—C3	125.1 (3)	C15—C16—H16	120.1
C6—C5—C4	121.9 (3)	C17—C16—H16	120.1
C6—C5—H5	119.0	C16—C17—C12	119.8 (3)
C4—C5—H5	119.0	C16—C17—H17	120.1
C5—C6—C7	120.1 (3)	C12—C17—H17	120.1
C5—C6—H6	120.0	C14—C18—H18A	109.5
C7—C6—H6	120.0	C14—C18—H18B	109.5
O3—C7—C8	117.1 (3)	H18A—C18—H18B	109.5
O3—C7—C6	122.6 (3)	C14—C18—H18C	109.5
C8—C7—C6	120.3 (3)	H18A—C18—H18C	109.5
C7—C8—C9	117.9 (3)	H18B—C18—H18C	109.5
C7—C8—C11	121.8 (3)	C1—O1—C9	121.4 (2)
C9—C8—C11	120.3 (2)	C7—O3—H3	109.5
O1—C9—C8	115.4 (2)	O5—C19—C20	113.8 (3)
O1—C9—C4	121.3 (3)	O5—C19—H19A	108.8
C8—C9—C4	123.3 (3)	C20—C19—H19A	108.8
C3—C10—H10A	109.5	O5—C19—H19B	108.8
C3—C10—H10B	109.5	C20—C19—H19B	108.8
H10A—C10—H10B	109.5	H19A—C19—H19B	107.7
C3—C10—H10C	109.5	C19—C20—H20A	109.5
H10A—C10—H10C	109.5	C19—C20—H20B	109.5
H10B—C10—H10C	109.5	H20A—C20—H20B	109.5
O4—C11—C12	122.8 (3)	C19—C20—H20C	109.5
O4—C11—C8	118.9 (2)	H20A—C20—H20C	109.5
C12—C11—C8	118.2 (2)	H20B—C20—H20C	109.5
C17—C12—C13	119.5 (3)	C19—O5—H5A	109.5
C17—C12—C11	120.7 (3)		
			
O2—C1—C2—C3	178.8 (3)	C3—C4—C9—C8	176.9 (3)
O1—C1—C2—C3	−1.5 (4)	C7—C8—C11—O4	−94.5 (3)
C1—C2—C3—C4	0.2 (5)	C9—C8—C11—O4	82.6 (3)
C1—C2—C3—C10	178.6 (3)	C7—C8—C11—C12	88.2 (3)
C2—C3—C4—C9	1.9 (4)	C9—C8—C11—C12	−94.7 (3)
C10—C3—C4—C9	−176.5 (3)	O4—C11—C12—C17	−179.6 (3)
C2—C3—C4—C5	−179.9 (3)	C8—C11—C12—C17	−2.4 (4)
C10—C3—C4—C5	1.7 (4)	O4—C11—C12—C13	−2.3 (4)
C9—C4—C5—C6	0.7 (4)	C8—C11—C12—C13	174.8 (2)
C3—C4—C5—C6	−177.6 (3)	C17—C12—C13—C14	1.8 (4)
C4—C5—C6—C7	0.4 (5)	C11—C12—C13—C14	−175.5 (3)
C5—C6—C7—O3	179.5 (3)	C12—C13—C14—C15	−2.6 (4)
C5—C6—C7—C8	−0.8 (4)	C12—C13—C14—C18	175.2 (3)
O3—C7—C8—C9	179.8 (2)	C13—C14—C15—C16	1.4 (5)
C6—C7—C8—C9	0.0 (4)	C18—C14—C15—C16	−176.3 (3)
O3—C7—C8—C11	−3.1 (4)	C14—C15—C16—C17	0.5 (6)
C6—C7—C8—C11	177.2 (3)	C15—C16—C17—C12	−1.3 (5)
C7—C8—C9—O1	−179.1 (2)	C13—C12—C17—C16	0.1 (5)
C11—C8—C9—O1	3.6 (3)	C11—C12—C17—C16	177.4 (3)
C7—C8—C9—C4	1.2 (4)	O2—C1—O1—C9	−179.6 (2)
C11—C8—C9—C4	−176.1 (2)	C2—C1—O1—C9	0.6 (4)
C5—C4—C9—O1	178.8 (2)	C8—C9—O1—C1	−178.2 (2)
C3—C4—C9—O1	−2.8 (4)	C4—C9—O1—C1	1.5 (4)
C5—C4—C9—C8	−1.5 (4)		

### Hydrogen-bond geometry (Å, °)

**Table:**

*D*—H···*A*	*D*—H	H···*A*	*D*···*A*	*D*—H···*A*
O3—H3···O5^i^	0.82	1.82	2.629 (3)	166.
O5—H5A···O2	0.82	1.95	2.764 (3)	169.
C17—H17···O2^ii^	0.93	2.54	3.398 (4)	154.
C20—H20B···O4^iii^	0.96	2.53	3.489 (5)	177.

Symmetry codes: (i) *x*, *y*−1, *z*; (ii) −*x*+1, *y*−1/2, −*z*+1/2; (iii) −*x*+2, −*y*+1, −*z*+1.
